# Solyntus, the New Highly Contiguous Reference Genome for Potato (*Solanum tuberosum*)

**DOI:** 10.1534/g3.120.401550

**Published:** 2020-08-05

**Authors:** Natascha van Lieshout, Ate van der Burgt, Michiel E. de Vries, Menno ter Maat, David Eickholt, Danny Esselink, Martijn P. W. van Kaauwen, Linda P. Kodde, Richard G. F. Visser, Pim Lindhout, Richard Finkers

**Affiliations:** * Plant Breeding, Wageningen University and Research, 6708 PB, Wageningen, The Netherlands; † Solynta, 6703 HA, Wageningen, The Netherlands; ‡ PepsiCo R&D, University of Minnesota, St. Paul, Minnesota 55108

**Keywords:** *Solanum tuberosum*, potato, diploid breeding, homozygosity, genome assembly

## Abstract

With the rapid expansion of the application of genomics and sequencing in plant breeding, there is a constant drive for better reference genomes. In potato (*Solanum tuberosum*), the third largest food crop in the world, the related species *S. phureja*, designated “DM”, has been used as the most popular reference genome for the last 10 years. Here, we introduce the *de novo* sequenced genome of Solyntus as the next standard reference in potato genome studies. A true *Solanum tuberosum* made up of 116 contigs that is also highly homozygous, diploid, vigorous and self-compatible, Solyntus provides a more direct and contiguous reference then ever before available. It was constructed by sequencing with state-of-the-art long and short read technology and assembled with Canu. The 116 contigs were assembled into scaffolds to form each pseudochromosome, with three contigs to 17 contigs per chromosome. This assembly contains 93.7% of the single-copy gene orthologs from the Solanaceae set and has an N50 of 63.7 Mbp. The genome and related files can be found at https://www.plantbreeding.wur.nl/Solyntus/. With the release of this research line and its draft genome we anticipate many exciting developments in (diploid) potato research.

Breeding of potato (*Solanum tuberosum*), the third largest food crop, has not resulted in significant genetic gain in yield when compared to other major crops (Douches *et al.* 1996; Duvick 2005; Rijk *et al.* 2013). Reasons for the limited genetic improvement in potatoes include a long generation cycle, polyploidy, heterozygosity and inbreeding depression (Lindhout *et al.* 2011). One method that has been used to study genetic effects is Genome Wide Associations Studies (GWAS), which have found major Quantitative Trait Loci (QTL) for important traits in tetraploid potatoes (Rosyara *et al.* 2016; Slater *et al.* 2016; Sverrisdóttir *et al.* 2017). However, more subtle or multiple QTL are difficult to detect using GWAS (Korte and Farlow 2013; Ott *et al.* 2015). Similarly, improvements seen by the use of a bi-parental cross in polyploids has been limited by uncertainties around the geno-phenotype correspondence, the partially informative markers determined for this cross, variations in meiotic mechanisms, outcrossing due to heterozygous genomic structure and how allelic and nonallelic combinations increase at an exponential rate with the number of alleles and thus only QTL with a larger effect can be identified (Li *et al.* 2012).

Recently, a method of hybrid breeding was newly applied to potato that is based on diploid homozygous inbred lines (Lindhout *et al.* 2011; Endelman and Jansky 2016; Su *et al.* 2020). Complex traits can be easily fixed in diploid inbreds, allowing for a more efficient selection and stacking of traits (Su *et al.* 2020). (Stockem *et al.* 2020) showed that experimental diploid hybrids were comparable in stability to tetraploid checks for different traits under field conditions, with some diploid hybrids yielding comparably to the worst performing checks. The genetic mapping of traits in diploid biparental segregating populations based on contrasting parents is more straightforward than in tetraploids because of the simpler genetics.

The Potato Genome Sequencing Consortium (Potato Genome Sequencing Consortium *et al.* 2011) has published the genome of the doubled monoploid *S. phureja* DM1-3 516 R44 (hereafter referred to as DM), which is a wild relative of the cultivated potato. Eventually, this lead to a reference with robustly oriented contigs along many unanchored super-scaffolds (Sharma *et al.* 2013). This first reference genome has proven very helpful for genetic studies. However, due to the limited read-lengths and high frequencies of repeated sequences in the potato genome, sequencing errors and assembly inaccuracies will have occurred. This fact, in combination with the large sequence diversion between DM and more commercially favorable potato genotypes (Uitdewilligen *et al.* 2013), have hampered detailed genome studies. To generate a “pure potato” reference line to facilitate genetic studies, we have developed a highly homozygous, vigorous and self-compatible diploid potato line, designated ‘Solyntus’. Moreover, we upgraded the sequencing approach to the state-of-the-art methods as of 2019: high coverage long read sequence technology in combination with ultra-deep short read sequencing, to generate a high quality *de novo* assembled reference genome.

To stimulate research on diploid potatoes, we are now releasing Solyntus and its sequence information as a universal research line. Here we present the first results of this sequencing project and the draft genome assembly.

## Methods & Materials

### Plant material

In 2008, Solynta initiated hybrid breeding in potato by making the first cross between a diploid potato and a self-compatible *S. chacoense* line. The first segregating population was grown in the field in 2010 (Lindhout *et al.* 2011). Most plants showed weak growth, poor flowering and produced only a few tubers and berries. After crosses, selections and many generations of selfing, highly homozygous inbred lines were generated. This process was regularly monitored using various types of DNA markers (Lindhout *et al.* 2018).

**Figure 1:**
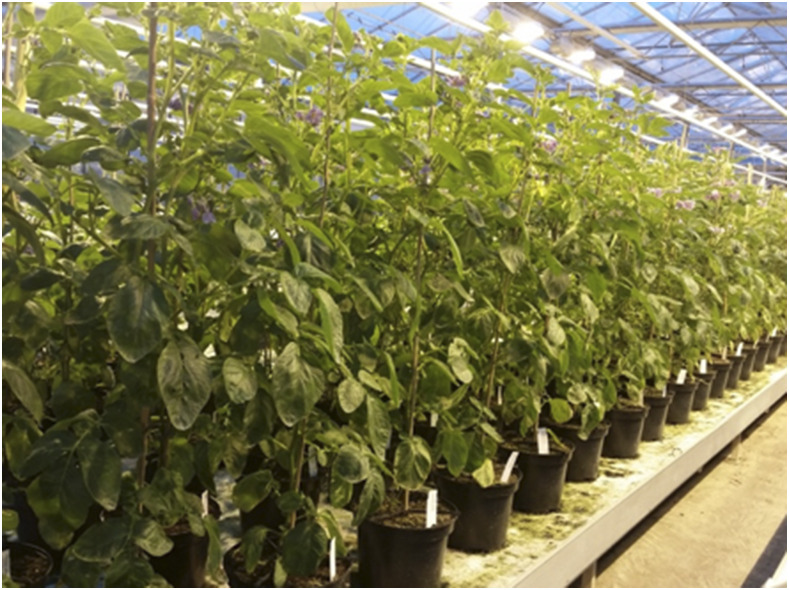
Pictures of Solyntus growing in the greenhouse.

**Figure 2:**
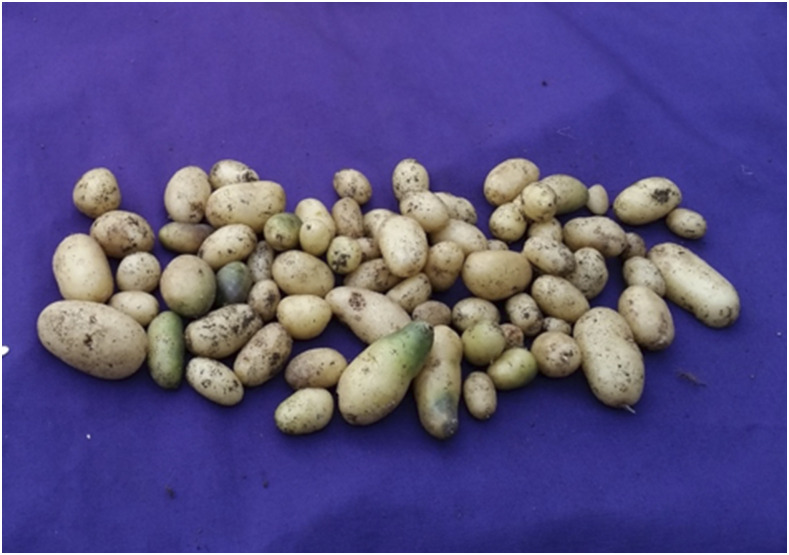
Picture of Solyntus tubers.

From this breeding population, which consisted of thousands of inbred lines, derived from various diploid potato sources as described by (Lindhout *et al.* 2018), a single F_9_ plant was selected. The features of this plant were that it: a) was phenotypically uniform and stable in greenhouse experiments as can be seen from Figure 1 and Figure 2, b) was growing vigorously, producing tubers, flowering profusely and setting seed both upon crosses as well as self-pollination, c) could be grown *in-vitro* on solid medium and regenerated from stem explants, d) had a high level of homozygosity and e) was phenotypically uniform over generations. Solyntus generates good tuber yields and numbers in the greenhouse. It produces round tubers with a creamy flesh.

### DNA extraction, library preparation and sequencing

The young leaves of three plants of the cultivar Solyntus we pooled into a single sample. High molecular weight DNA was extracted from this sample according to (Bernatzky and Tanksley 1986). Library preparation, excluding the DNA fragmentation step, was performed using the SQK-LSK109 Ligation Sequencing kit (Oxford Nanopore Technologies; Oxford, UK) according to the instructions. Long read data were generated using an Oxford Nanopore GridION using 10 flowcells and a run-time of 48 hr. Quality control was performed on the long reads using minIONQC (Lanfear *et al.* 2019) and NanoComp (De Coster *et al.* 2018). Adaptors were removed using Porechop (Wick 2018) and the data were filtered with Filtlong (Wick 2019), removing the worst 10% of read bases while prioritizing read length.

Furthermore, 4.6 ug of DNA was sent to the University of Minnesota Genomic Center to generate three TruSeq DNA PCR free libraries of 450bp inserts (Illumina Inc; San Diego, USA). These libraries were subsequently pooled and sequenced across two lanes to generate 250 bp paired-end reads on the Illumina NovaSeq platform. The short read data quality was evaluated with FastQC (Andrews S 2018) and an assessment of the genome characteristics was made using k-mer counts, Jellyfish (k = 71) (Marçais and Kingsford 2011) and GenomeScope (Vurture *et al.* 2017).

### Genome assembly and scaffolding

Long reads, with a minimum read length of 15 Kbps, were used for assembly by Canu v1.8 (Koren *et al.* 2017) with a maximum coverage correction value set to 200, maximum overlap error rate set to 0.15 and an estimated genome size of 850 Mbps. Purge Haplotigs (Roach *et al.* 2018), including the optional “trimming of overlapping contig ends” step, was used to flatten regions of heterozygosity into a single consensus sequence. The contigs were then polished with two iterations of Pilon v1.23 (Walker *et al.* 2014) using the Illumina reads. Finally, RaGOO v1.1 (Alonge *et al.* 2019) was used for reference guided scaffolding of the contigs using DM v4.03 (Potato Genome Sequencing Consortium *et al.* 2011; Sharma *et al.* 2013) as the reference.

### Genome analysis and quality assessments

QUAST v5.0.2 (Gurevich *et al.* 2013) was used to determine the basic characteristics of the assembly. Seperately, completeness of the Solyntus v1.1 assembly was assessed using BUSCO v4.0.5 (Simão *et al.* 2015).

Gene annotation was inferred from the annotations of potato DM v4.03 (Potato Genome Sequencing Consortium *et al.* 2011; Sharma *et al.* 2013) and tomato ITAG 4.0 (Hosmani *et al.* 2019) using GeMoMa v1.6.1 (Keilwagen *et al.* 2016, 2018). GeMoMa uses the gene annotations of a reference genome to predict protein-coding genes in a target assembly.

Illumina PE reads were mapped to the Solyntus v1.1 genome sequence using Minimap2 v2.14-r883 (Li 2018), had variants called with FreeBayes v1.3.2-38-g71a3e1c (Garrison and Marth 2012) to identify regions of heterozygosity and record the genome coverage in 30Kbps windows using Mosdepth (Pedersen and Quinlan 2018). From there we plotted the variation in coverage and heterozygous SNPs across the genome using Circos v0.69-8 (Krzywinski *et al.* 2009) as well as the mapped contigs to the final pseudochromosomes in alternatingly colored blocks.

D-GENIES v1.2.0 (Cabanettes and Klopp 2018) was used to visualize the dot plot relations between Solyntus v1.1 and DM v4.03 using Minimap2 (Li 2018) and default settings.

### Data availability

The final assembly and annotation files are available on https://www.plantbreeding.wur.nl/Solyntus/ for download and in a Genome Browser. In addition, data including the genome sequence and raw sequencing reads have been deposited to NCBI under BioProject ID PRJNA631911. Analysis files including the BUSCO, Quast and Circos output files are available in the supplementary data. The biological material of Solyntus is available for scientific research under an MTA and can be requested from Solynta. It is already being used by a dozen academic groups (Lin *et al.* 2020) to perform research aimed at increasing fundamental knowledge in potato. Supplemental material available at figshare: https://doi.org/10.25387/g3.12288152.

## RESULTS & DISCUSSION

### Raw sequence quality

Initially, 6 917 092 long reads, totaling 71.9 Gb of data, were obtained using the Oxford Nanopore GridION platform. After removing adapters with Porechop (Wick 2018) and filtering with Filtlong (Wick 2019), 3 735 580 sequences were left with a mean length of 4 996 bps and length ranging from 4 646 – 420 405 bps and a GC content of 35% according to NanoComp (De Coster *et al.* 2018).

Illumina NovaSeq sequencing provided us with 922 636 449 pairs of 250 bp sequence reads. The GC content was 35% with an average sequence quality phred score of 36 according to FastQC (Andrews S 2018).

### Genome size and characteristics

**Figure 3:**
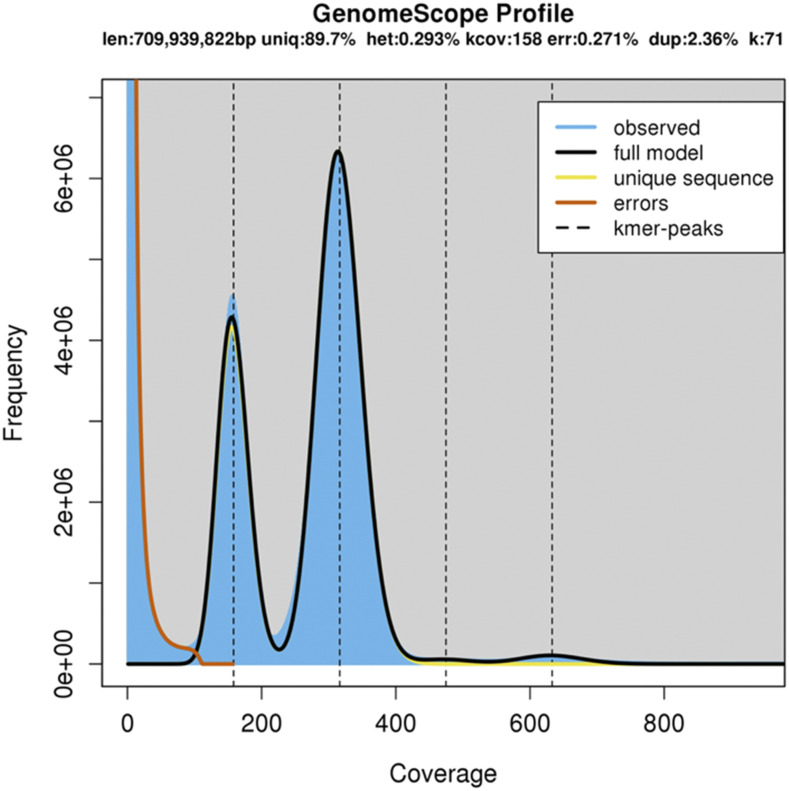
A K-mer (K = 71) distribution based on the high coverage Illumina reads as modeled and visualized by genomescope, with a maximum read count cut-off of 2500.

In order to assess the size and residual heterozygosity of the genome, the Illumina reads were used to count k-mers (K = 71) using Jellyfish (Marçais and Kingsford 2011) and analyzed using GenomeScope (Vurture *et al.* 2017). The GenomeScope analysis (Figure 3) reports an estimated genome size of 710 Mbps with 0.3% of the genome estimated to be heterozygous and 89.7% of the genome unique. Solyntus has been inbred for nine generations, so a high level of homozygosity was expected. Given the large portion of the genome found to be unique, we expect that the remaining heterozygosity in Solyntus will be localized to a few regions in the genome, and that the majority of the genome is homozygous. Therefore we can assemble much of the genome with a strategy tailored to homozygous genomes.

### Genome assembly and quality

Initially, the Canu (Koren *et al.* 2017) assembly started with 1 433 419 reads, totalling 44.7 Gb and with a length of over 15 Kbps, which provided a coverage of 52.66 times the estimated genome size (850 Mb). After error correction and trimming, 1 177 677 reads, totalling 38.4 Gb remained for assembly. This resulted in 661 contigs with a total length of 894 327 336 bps (including 64 repeats covering 11 873 955 bps).

Purge Haplotigs (Roach *et al.* 2018) was used to flatten the assembly. Purge Haplotigs identifies repeats and contigs containing a second haplotype, refered to as haplotigs, by first looking at the read depth and then the alignment scores using Minimap2 (Roach *et al.* 2018). Contigs where 80% of the sequence has a coverage much higher or lower than expected are marked as junk (Roach *et al.* 2018). An expanded Oxford Nanopore Technologies (ONT) dataset of approximately 70 Gb was mapped to the genome using minimap and a coverage histogram was generated. This resulted in a read depth histogram, with the homozygous peak at a mean coverage of 129x and a heterozygous peak at a mean coverage of 65x. Based on the distribution around these two peaks, a value of 35 was selected as the low read depth cutoff, a value of 94 as the low point between the haploid and diploid peaks and a value of 200 as the high read depth cutoff. Contigs with an alignment score greater or equal to 80% were marked as haplotigs while those greater or equal to 250% are marked as repeats (Roach *et al.* 2018). Of the 661 contigs produced by Canu, Purge Haplotigs classified and removed 438 contigs as repeats, 52 contigs as haplotigs and 54 contigs as junk. In the final trimming of the overlapping-contig-ends step, one additional contig was removed because it overlapped with 7 other contigs, resulting in a final assembly of 116 contigs.

Subsequently, the 116 contigs were polished twice using Pilon (Walker *et al.* 2014) and assembly statistics were determined using QUAST (Gurevich *et al.* 2013). The assembly had a total length of 716 161 047 bps and the length of the shortest contig at 50% of the total genome length (N50) was 13 367 893 bps (TABLE 1). RaGOO (Alonge *et al.* 2019) was then used to scaffold the assembly into 12 pseudochromosomes based on the DM v4.03 pseudochromosomes (Potato Genome Sequencing Consortium *et al.* 2011; Sharma *et al.* 2013), with all 116 contigs placed on these pseudochromosomes. A pseudochromosome minimally consisted of 3 contigs (StSOLv1.1ch11 and StSOLv1.1ch12; TABLE 2) and maximally of 17 contigs (StSolv1.1ch08; TABLE 2) demonstrating the high continuity of the assembly. The N50 and the smallest number of contigs whose length sum makes up 50% of the genome size (L50) of the final scaffolded assembly were 63 701 590 bps and 6 respectively. The final assembly also had an average of rate of 1.45 uncalled bases (Ns) per 100 kbps (TABLE 1).

**Table 1:** Solyntus de Novo Genome Assembly Metrics Estimated Using QUAST

	Contigs	Pseudomolecules
Number of Contigs/Scaffolds:	116	12
Largest Contig/Scaffold:	44 448 130	72 008 707
Total Length:	716 161 047	
N50:	13 367 893	63 701 590
N75:	7 229 460	57 022 023
L50:	15	6
L75:	34	9
Number of Ns per 100 Kbps:	0	1.45
GC Content (%):	34.82	

**Table 2:** Summary of the Number of Contigs Placed by RaGOO into Each Pseudochromosome

Chromosome	Number of Contigs Placed	Total Length
StSOLv1.1ch01	16	59 557 243
StSOLv1.1ch02	7	42 706 079
StSOLv1.1ch03	8	63 701 590
StSOLv1.1ch04	12	72 008 707
StSOLv1.1ch05	8	58 542 029
StSOLv1.1ch06	7	65 489 876
StSOLv1.1ch07	10	41 124 029
StSOLv1.1ch08	17	67 850 527
StSOLv1.1ch09	12	70 978 473
StSOLv1.1ch10	13	49 170 357
StSOLv1.1ch11	3	57 022 023
StSOLv1.1ch12	3	68 020 514

BUSCO is a set of universal single-copy orthologs used to determine the completeness of the genome. Using the obd10 Solanaceae set, 93.7% of the orthologs could be identified within our assembly (Simão *et al.* 2015) (TABLE 3).

**Table 3:** Output from BUSCO Analysis Pipelines to Assess Genome Completeness

	Solanaceae (odb10)
Complete BUSCOs:	2859 (93.7%)
Complete and Single Copy BUSCOs:	2771
Complete and Duplicated BUSCOs:	88
Fragmented BUSCOs:	91
Missing BUSCOs:	102
Total BUSCO groups searched:	3052

### Genome annotation

Using the DM v4.03 genome annotation set (Sharma *et al.* 2013), 62 322 features were predicted using a homology-based approach (Keilwagen *et al.* 2016, 2018). Additionaly, using the ITAG 4.0 genome annotation set (Hosmani *et al.* 2019), 35 456 features were predicted using the same homology-based approach. This tomato based set was added because of genes such as the OFP20 ortholog, which has been observed in M6 (Leisner *et al.* 2018) and Solyntus but not DM (Wu *et al.* 2018). The gene identifiers match with the original datasets for easy reference. These two annotation sets are meant as a starting point for further research and curation into the gene space of Solyntus.

### Determine heterozygous regions

**Figure 4:**
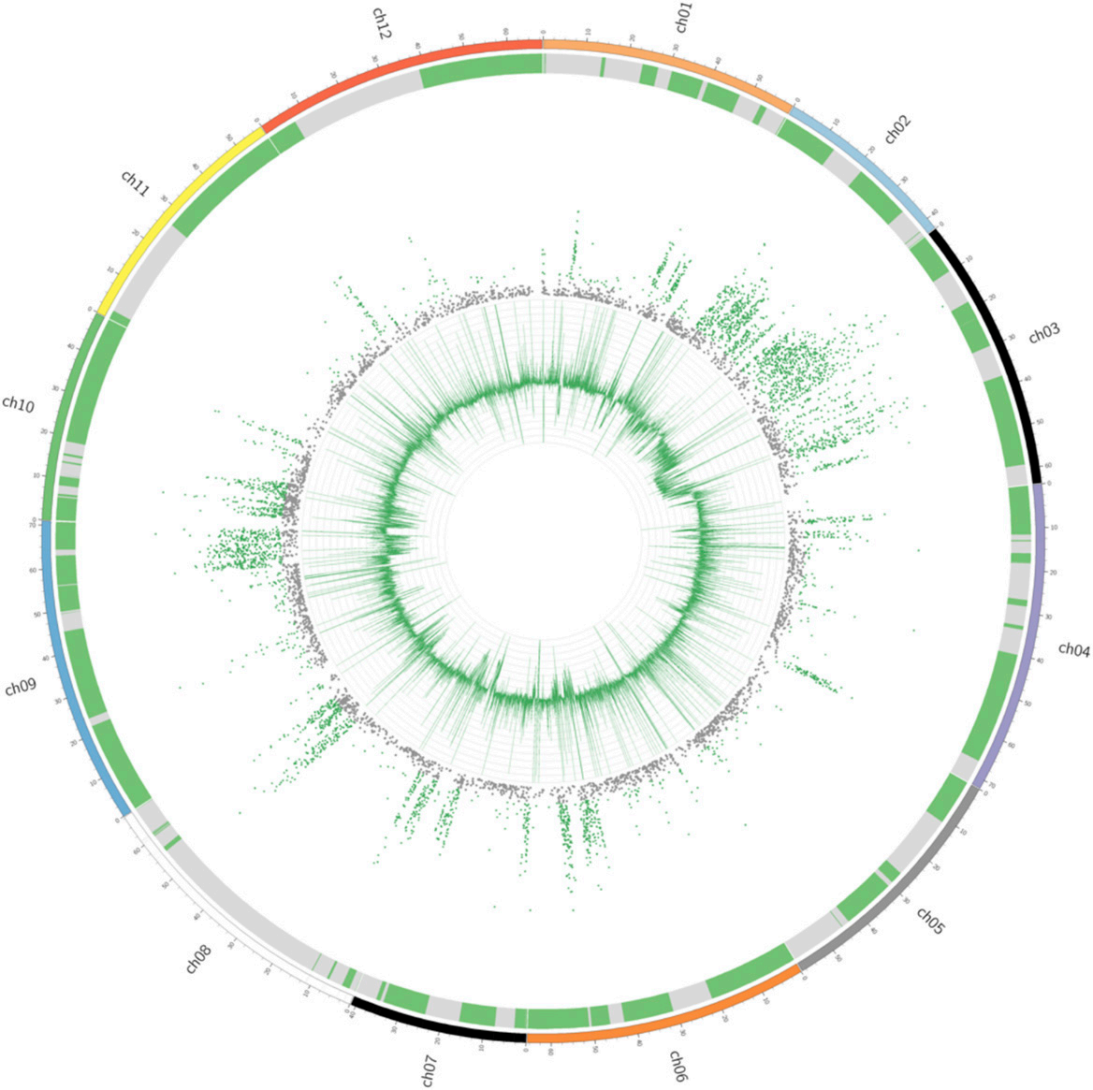
Circos plot that shows Solyntus assembly (outer ring), heterozygous SNP rate of the short reads (middle ring) and the coverage of the short reads per bin (innermost ring). The alternating green and gray blocks of the outer ring symbolize the contigs as they were placed into pseudochromosomes.

Genomescope analysis already indicated residual heterozygosity in the genome. To further investigate this, the Illumina reads were mapped against the genome and variants were determined. Genome coverage and SNP frequency in 30 Kbp bins were subsequently calculated and visualized using a circos plot (Figure 4).

The outermost ring shows each chromosome in the Solynstus v1.1 assembly in a variety of colors. The second most outer ring shows the contigs that make up each chromosome (in alternating colors). The middle ring shows the heterozygous SNPs between the Solyntus reference and mapped regions (colored green when above the baseline SNP rate of 200 SNPs per 30Kb; determined by visual inspection of the circos plot). Finally, the innermost ring demonstrates the coverage of the short reads against the reference in 30 Kbp windows.

By considering a SNP rate of 200 SNPs per 30 Kbp as a threshold for heterozygosity, of the total 21 776 windows, 4 379 windows showed a signal above this threshold, which is equivalent to 20.1% of the genome still being heterozygous. This number is much higher than the number expected for an F9 inbred. This may be due to a combination of unnoticed and undesired outcrossing during the generation of Solyntus and a preferential selection of heterozygotes in the inbreeding process due to inbreeding depression. Remaining heterozygosity in M6 was posited as a possible effect of lethal or deleterious alleles being maintained in repulsion with beneficial alleles (Jansky *et al.* 2016; Leisner *et al.* 2018), but there is no indication of such lethal alleles in Solyntus as, in the selfed siblings of Solyntus, individuals were always detected that showed homoygosity for these heterozygous regions (not shown). The authors of the M6 paper also suggested that higher homozygosity would be difficult to achieve as regions of reduced recombination contain a higher rate of deleterious alleles and thus require sexual propogation to purge (Leisner *et al.* 2018).

### Comparison of Solyntus v1.1 vs. DM v4.03 Genome Sequence

**Figure 5:**
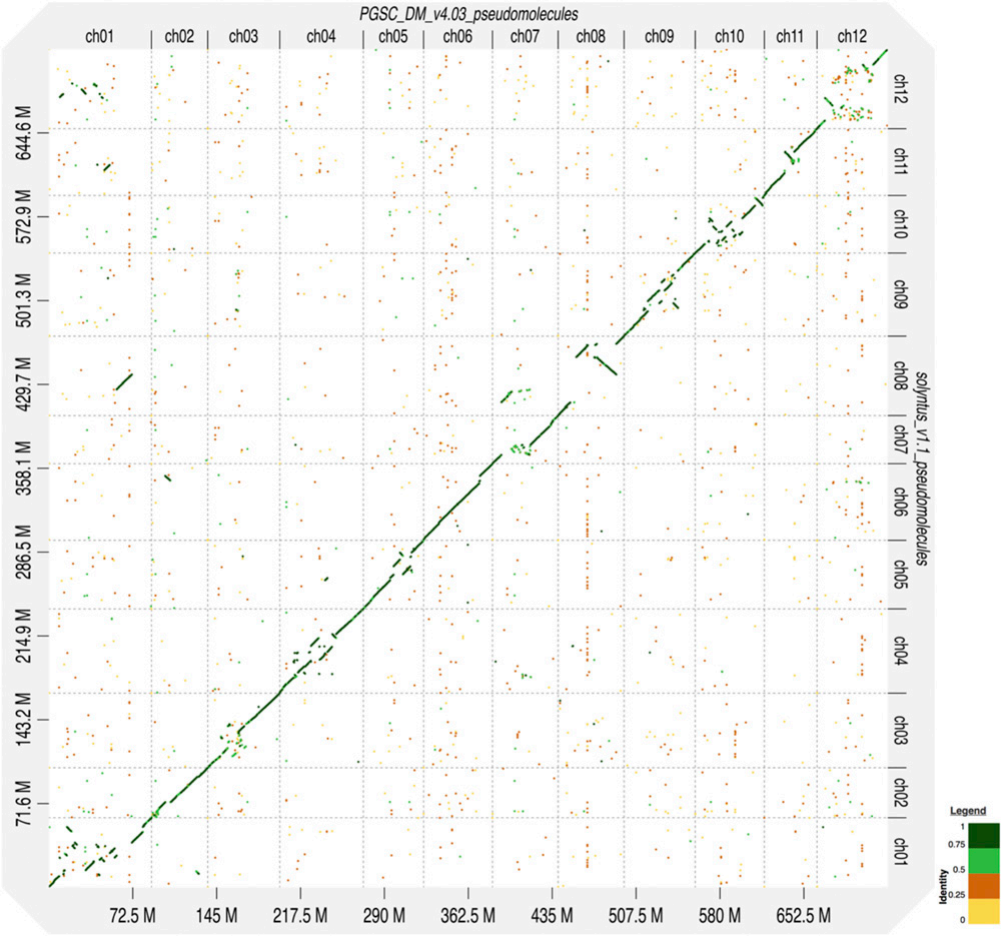
Identity plot between Solyntus V1.1 and DM V4.03 pseudochromosomes using D-GENIES with noise filtered out.

The pseudomolecule representations of Solyntus v1.1 was compared to the pseudomolecule ordering of DM v4.03 using a dotplot strategy by D-GENIES (Cabanettes and Klopp 2018) (Figure 5). As RaGOO was used to order the Solyntus contigs into pseudomolecules based on DM v4.03 as a reference, this might introduce errors into the orientation of the Solyntus v1.1 assembly. However, as the majority of the contigs were already very large and the developed pseudomolecules consisted of only a limited set of contigs (between 3 to 17; TABLE 2), we were able to reduce the risk of orientation errors and use this strategy to highlight the differences in sequence ordering between the assemblies within the individual contigs. We describe here three cases between the assemblies highlighting some of the observations we made:

There are inversions between the two, such as on chromosome 11. This chromosome was assembled from 3 contigs (TABLE 2) including one that spans both the middle and upper portions of the chromosome, though only the center region maps inversely to the corresponding region of DM.

There are also situations of divergence between the two, such as on chromosome 12. There, the assembly was made from only 3 contigs (TABLE 2) that span the whole chromosome and the identity at the edges was found to be very high and linear but the sequences strongly diverge toward the center of the chromosome, where a high density of repeats is present. It is most likely that DM, with it’s shorter read lengths, struggled to correctly assemble the centromeric region with it’s high concentration of repeats, though rapid centromeric development has also been observed in potato (Gong *et al.* 2012) and could point to a biological difference. To determine the root cause of this divergence, longer read sequencing of DM in this area would be required.

There are also indications of a translocation or misassembly in one of the genomes based on one contig in chromosome 8. While it was placed on StSOLv1.1ch08 of the Solyntus assembly, the first tenth of the contig maps best with a segment of ST4.03ch07 on DM, the next third of the contig has a high identity with ST4.03ch01 on DM and it is only about the last ∼20 Mbps that map best to ST4.03ch08.

Finally we also compared the STc4.03ch00 of DM with Solyntus v1.1 to see if we could place some of these previously unanchored sequences. Selecting only the sequences over 50 000 bps, the unanchored sequences aligned predominantly to the middle of chromosomes StSOLv1.1ch01, StSOLv1.1ch03, StSOLv1.1ch05, StSOLv1.1ch06, StSOLv1.1ch09, and StSOLv1.1ch11 (not pictured).
